# Drug lag and associated factors of orphan drugs approved by the U.S. in China

**DOI:** 10.3389/fphar.2025.1595497

**Published:** 2025-08-29

**Authors:** Qihui Mo, Jiaqi He, Qixiang Guo, Yue Yang

**Affiliations:** ^1^ School of Biomedical Engineering, Hainan University, Sanya, China; ^2^ School of Business Administration, Shenyang Pharmaceutical University, Shenyang, China; ^3^ School of Pharmaceutical Sciences, Tsinghua University, Beijing, China; ^4^ Key Laboratory of Innovative Drug Research and Evaluation, National Medical Products Administration, Beijing, China

**Keywords:** orphan drug, launch delay, clinical trial, drug access, expedited program

## Abstract

**Background:**

A pronounced disparity exists in the accessibility of orphan drugs between China and developed countries, such as the United States. Identifying and analyzing the critical determinants that contribute to this gap is essential for enhancing the availability of orphan drugs and promoting equitable access for patients.

**Methods:**

We included all new orphan drugs approved by the US Food and Drug Administration (FDA) between 2013 and 2023 and collected their approval information in China and the United States. Major factors of interest included accelerated review pathway, locations where pivotal clinical trials were conducted, therapeutic category, and other factors affecting the delay in drug launch. They were analyzed using multinomial logistic regression, analysis of covariance, and the Mann-Whitney U test.

**Results:**

The FDA approved a total of 242 new orphan drugs between 2013 and 2023. Among these, 119 (49.2%) of these drugs had been approved in China as of 1 January 2025, with a median lag time of 1,004 days (2.75 years). Among them, 47 drugs (41.2%) were included in the China’s List of Rare Diseases. Multinomial logistic regression analysis revealed that the conduct of pivotal trials supporting FDA approval in mainland China was associated with whether such drugs were launched in China (odds ratio = 10.53, 95% confidence interval 3.67–40.79; P < 0.001). Furthermore, the Mann-Whitney U test indicated that such characteristics as the inclusion of indications in China’s List of Rare Diseases, the granting of breakthrough therapy designation by the National Medical Products Administration (NMPA), and the inclusion of pivotal clinical trials in mainland China were all associated with the shortening of drug lag time.

**Discussion:**

Our findings suggest that orphan drug approval delays in China have improved significantly, but still face significant challenges. China’s involvement in the global collaborative development of drugs not only helps shorten the relative lag time for drugs to obtain approval in China but also avoids repeated trials and significantly improves R&D efficiency. We recommend that pharmaceutical companies include Chinese patients in the drug development stage so that they can enjoy cutting-edge innovative therapies more quickly.

## 1 Introduction

Developing and ensuring access to orphan drugs is a critical global public health issue. Orphan drug development encounters unique challenges, including small patient populations and the difficulty of recruiting patients for clinical trials. National drug regulatory agencies’ approval policies not only dictate market access to drugs but also impact treatment opportunities and quality of life for those with rare diseases. However, due to varying standards and processes across different agencies for assessing safety and efficacy, global drug approval dates differ significantly (Enya et al., 2025).

Drug lag is defined as the delay between the global first approval (usually from the FDA or the European Medicines Agency (EMA)) and the regulatory approval from the national health authority in each country (Andersson, 1992). Due to differences in regulatory policies across various regions, the drug lag phenomenon is particularly evident in the field of new drugs and even more pronounced in the field of orphan drugs. Cao et al. analyzed the imported rare disease drugs approved by the National Medical Products Administration (NMPA) in 2022, finding a time difference of about 3 years between domestic and foreign market launches (Cao et al., 2024). Additionally, Zhu et al. analyzed 135 innovative drugs approved by the NMPA from 2012 to 2019 that the FDA had already approved. The average lag time in China was 1,274 days (3.5 years), with the delay for orphan drugs being particularly significant (Zhu and Chen, 2024).

The situation facing orphan drug patients, where drugs are available overseas but not approved domestically, is particularly prominent in China. Despite the huge scale of the Chinese pharmaceutical market, the R&D and marketing of innovative drugs, particularly orphan drugs, still face many challenges.

In recent years, China has gradually improved its approval policies to promote orphan drug R&D. Orphan drugs are eligible for priority review procedures and market exclusivity periods, and priority in national medical insurance access negotiation (Liu et al., 2024).

However, compared with the United States, China’s orphan drug policy framework remains in a developmental phase, and the efficiency of the approval process requires further optimization.

Under the Orphan Drug Act of 1983, the United States defines a rare disease as one with a prevalence of fewer than 200,000 affected individuals annually (Ruiz et al., 2023). China has not yet established a definition based on prevalence and the number of patients but has instead identified rare diseases by list. In 2018 and 2023, the National Health Commission (NHC) successively released two batches of rare disease lists, which included a total of 207 rare diseases (China National Health Commission, 2018).

China has a huge orphan drug market. It is estimated that there are approximately 20 million to 54 million rare disease patients in China. (Zhao, 2022). However, based on the indications in the China’s List of Rare Diseases (CLRD), there are still nearly 30 list diseases that face the current situation of drugs available overseas but not approved domestically (Liu et al., 2023; Zhi et al., 2023). The treatment needs of many rare disease patients remain unmet.

In recent years, China has adopted a series of policies to accelerate market access for orphan drugs and other urgently needed drugs. The 2020 new version of the “Administrative Measures for Drug Registration” includes innovative drugs for the prevention and treatment of rare diseases in the priority review and approval procedure, and sets a 70-day review time limit for orphan drugs that are urgently needed in clinical trials but are not yet marketed in China (China NMPA, 2020; Wei et al., 2025). In 2017, China implemented the “General Principles for the Planning and Design of Multiregional Clinical Trials” issued by The International Council for Harmonisation of Technical Requirements for Pharmaceuticals for Human Use (ICH), This initiative enhanced cooperation between domestic and international pharmaceutical companies and improved the acceptance of the results of MRCTs results (China NMPA, 2017).

In 2018 and 2020, the NMPA issued two guidelines: the “Technical Guidelines for Accepting Overseas Clinical Trial Data” and the “Clinical Technical Requirements for Drugs Marketed Overseas but Not Marketed in China”. These guidelines indicate that overseas clinical trial data can be used to support drug registration applications in China if the data demonstrate efficacy and safety and do not exhibit racial sensitivity. Under these conditions, domestic clinical trials may be exempted (Tang et al., 2021).

These policies reflect China’s high degree of recognition of clinical trial data from overseas or MRCTs, particularly for rare diseases. This open attitude helps to reduce the time lag between the launch of orphan drugs abroad and in China, and thereby addressing the unmet clinical needs of rare disease patients in the country (Liu et al., 2024; Qiao et al., 2022).

Existing literature has studied drug approval delays in China, but mostly focuses on innovative and oncology drugs, with little analysis of orphan drug delays (Luo et al., 2022; Luo et al., 2023). This study aims to explore the current situation and influencing factors of the delay in the approval of orphan drugs in China compared with the United States, to narrow the gap in drug accessibility between China and the United States. A deeper understanding of the impact of the regulatory system on orphan drug accessibility is of great significance for improving China’s orphan drug policy and improving patients’ access to medicines.

## 2 Materials and methods

### 2.1 Sample identification

**FIGURE 1:**
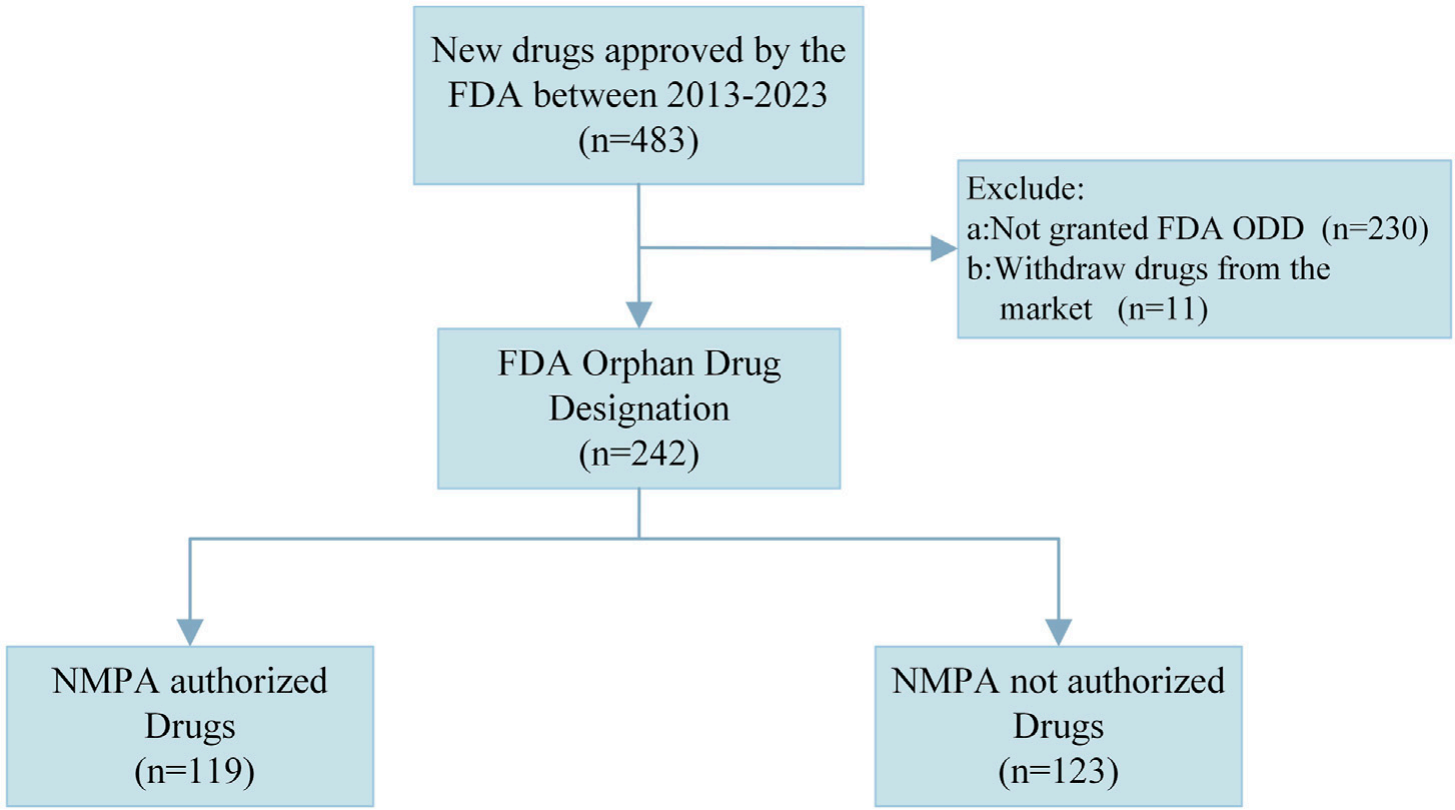
Flowchart of sample identification. NMPA, National Medical Products Administration, FDA, Food and Drug Administration; ODD, Orphan Drug Designation.

The study covers orphan drugs approved by the FDA from 1 January 2013 to 31 December 2023 in the United States. Figure 1 shows a flowchart for the sample identification process. We excluded drugs that were not designated as orphan drugs by the FDA and those that have been withdrawn from the U.S. and EU markets. Ultimately, a total of 242 drugs were analyzed in the study.

### 2.2 Data collection and definitions

Based on the Drugs @FDA database (U.S. Food and Drug Administration) and the Insight public database, we collected all new chemical entities and new biological products approved by the FDA between 1 January 2013 and 31 December 2023. For each drug, we collected basic information, including the FDA approval date, registration class (New Drug Application or Biologics License Application), orphan drug designation, expedited review pathway (Priority review, Accelerated approval, Fast track, and Breakthrough therapy), whether it is an multi-regional clinical trials (MRCTs) in a pivotal clinical trial, the nationality of the Marketing Authorization Holder (MAH), and company size classified by market capitalization (small: <$1 billion, medium: $1 billion to <$8 billion, large: ≥$8 billion) (Enya et al., 2025), and approved indications. We then classified these drugs according to the WHO’s Anatomical Therapeutic Classification (ATC).

To analyze the impact of conducting pivotal clinical trials in different regions and including diverse populations on drug lag, we categorized the trial sites into three groups: those located in the Chinese mainland, those in Taiwan and/or Hong Kong (China), and those without any Chinese research centers.

We searched the FDA Orphan Drug Designation and Approval Database to determine whether each drug had received orphan drug designation. We also used the CLRD by China’s National Health Commission and the Insight database to identify whether the indications of orphan drugs approved in the United States are included in CLRD. In addition, we collected information on the clinical trials of these drugs from ClinicalTrials.gov and the FDA’s “Drug Trial Snapshot”, and drug review reports.

In this study, drug lags were classified as absolute and relative lags (Andersson, 1992). Absolute lag is defined as the number of orphan drugs that have not been approved by the NMPA within the same period. In addition, relative lag is defined as the time difference between the first approval of an orphan drug in China and its first approval in the United States. Relative lag is calculated as the marketing approval dates by the FDA minus the approval dates by the NMPA.

### 2.3 Statistical analysis

In this study, we used multinomial logistic regression to identify factors influencing whether drugs approved in the United States will be marketed in China, The dependent variables include therapeutic class, therapeutic area, company size, FDA accelerated pathways (Breakthrough Therapy Designation, Fast Track and Accelerated Approval), whether the pivotal clinical trial is MRCTs, and pivotal clinical trial locations that supported the FDA approval.

We used the Mann-Whitney U test and analysis of covariance to identify factors associated with the relative lag of drugs. Considering that the lag time may be caused by delays in clinical development or long review times in China, we hypothesized that it may be affected simultaneously by the design of the clinical trial and the review pathway of the regulatory agency, enterprises’ development strategies.

For clinical trial design, we selected two independent variables: MRCTs and the locations of a pivotal trial. For expedited approval programs, we selected Breakthrough Therapy Designation and Accelerated Approval by the FDA and Breakthrough Therapy Designation and Conditional Approval by NMPA, as they represent accelerated approval programs for new drug development. The CLRD were also included as independent variables, because drugs listed therein receive preferential treatment in the review process. In addition, we also considered the nationality of MAH and company size.

All statistical analyses were performed using R (version 4.5.0) and GraphPad Prism (version 9.5). Statistical significance was set at P < 0.05.

## 3 Results

### 3.1 Orphan drug lag in China

A total of 242 new drugs granted orphan drug status were approved by the FDA between 2013 and 2023. Their characteristics are shown in Table 1. As of 15 February 2025, among these drugs, 119 (49.2%) have been approved in China, and 123 (50.8%) have not been approved. Among the drugs that have not been authorized in China, 67 (54.5%) have indications listed in CLRD, and 23 (34.3%) of these have indications with no alternative treatment in China.

**TABLE 1:** Sample characteristics.

Variables	All drugs N (%)	Drug approved in China N (%)
Total	242 (100)	119 (49.2)
Type of drug		
Chemical drugs	155 (64.0)	77 (49.7)
Biologics	87 (36.0)	42 (48.3)
Therapeutic area		
Oncology Drugs	103 (42.6)	62 (60.2)
Non-oncology Drugs	139 (57.4)	57 (41.0)
China’s List of Rare Diseases		
Yes	114 (47.1)	47 (41.2)
No	128 (52.9)	72 (56.2)
Urgently needed overseas drugs		
Yes	23 (9.5)	17 (73.9)
No	219 (90.5)	102 (46.6)
Expedited pathway in US		
Priority Review	190 (78.5)	92 (48.4)
Fast Track	117 (47.1)	48 (42.1)
Accelerated Approval	69 (28.5)	41 (59.4)
Breakthrough Therapy	118 (48.8)	64 (54.2)
Pivotal Trial as MRCTs in FDA		
Yes	201 (83.1)	109 (54.2)
No	41 (16.9)	10 (24.4)
Pivotal trial locations in FDA		
No China sites	173 (71.5)	70 (40.5)
With sites in China mainland	34 (28.5)	31 (91.2)
With sites in Hongkong/Taiwan, China	54 (22.3)	36 (66.6)
FDA review times, median (IQR), days	243 (214-334)	242 (184-329)
NMPA review times, median (IQR), days	NA	371 (300-497)
Launching lag time, median (IQR), days	NA	1,004 (723-1,442)
MAH in the United States		
Large pharma	137 (56.6)	75 (54.7)
Small to mid-sized enterprise	105 (43.4)	44 (41.9)
ATC		
A (Alimentary tract and metabolism)	27 (11.2)	7 (25.9)
B (Blood and blood forming organs)	15 (6.2)	6 (40.0)
C (Cardiovascular system)	7 (2.9)	4 (57.1)
D (Dermatology)	1 (0.4)	0 (0)
G (Genito-urinary system and hormones)	0 (0)	0 (0)
H (Systemic hormonal preparations, excluding sex hormones)	4 (1.7)	2 (50.0)
J (Anti-infectives for systemic use)	12 (5.0)	6 (50.0)
L (Antineoplastic and immunomodulatory agents)	126 (52.1)	76 (60.3)
M (Musculoskeletal system)	12 (5.0)	4 (33.3)
N (Nervous system)	19 (7.9)	9 (47.4)
P (Antiparasitic products, insecticides and repellents)	6 (2.5)	0 (0)
R (Respiratory system)	6 (2.5)	3 (50.0)
S (Sensory organs)	2 (0.8)	1 (50.0)
V (Various)	5 (2.1)	1 (20.0)

China’s List of Rare Diseases, In 2018 and 2023, the National Health Commission (NHC) released two batches of the “Rare Disease Catalogue”, which included a total of 207 rare diseases; Urgently needed overseas drugs, The National Medical Products Administration has successively released three lists of urgently needed overseas new drugs for clinical use, with a total of 73 varieties, of which more than half are rare disease treatments. These drugs can enjoy a dedicated channel for expedited review; MNC, multi national company; MRCTs, Multi-regional Clinical Trials; ATC, Anatomical Therapeutic Chemical (ATC) classification.

The absolute lag for NDAs and BLAs were close (49.7% vs 48.3%). Among all the drugs approved by the FDA, 114 (47.1%) have indications that are included in CLRD and 47 (41.2%) of these have been approved for marketing in China. Additionally, among the 23 (9.5%) drugs approved by the FDA and classified as UNODs, 17 (73.9%) have already been authorized in China.

From the perspective of expedited approval programs, drugs with Breakthrough Therapy status and Accelerated Approval status from the FDA had a higher percentage of approvals in the NMPA, at 54.2% and 59.4%, respectively. These rates are significantly higher than those for the other two accelerated review pathways.

According to the ATC categories, antineoplastic and immunological drugs had the lowest absolute lag (60.3%), while dermatology, genitourinary and hormones, antiparasitic drugs, insecticides, and repellents were not approved in China.

Further analysis of the characteristics of pivotal clinical trials that supported FDA approval shows (Table 1) that the proportion of pivotal clinical trials for drugs approved by the FDA that are MRCTs is 201 (83.1%), and 109 (54.2%) have been approved in China, accounting for 92.4% of the total number approved in China.

Of all drugs approved by the FDA, 173 (71.5%) had pivotal clinical trials that did not include China as a trial site. Among these drugs, only 70 have been approved for marketing by the NMPA, accounting for 40.5%. In contrast, 34 (28.5%) drugs included the Chinese mainland as a clinical trial center, and the approval rate of these drugs by the NMPA was significantly higher (91.2%).

**FIGURE 2:**
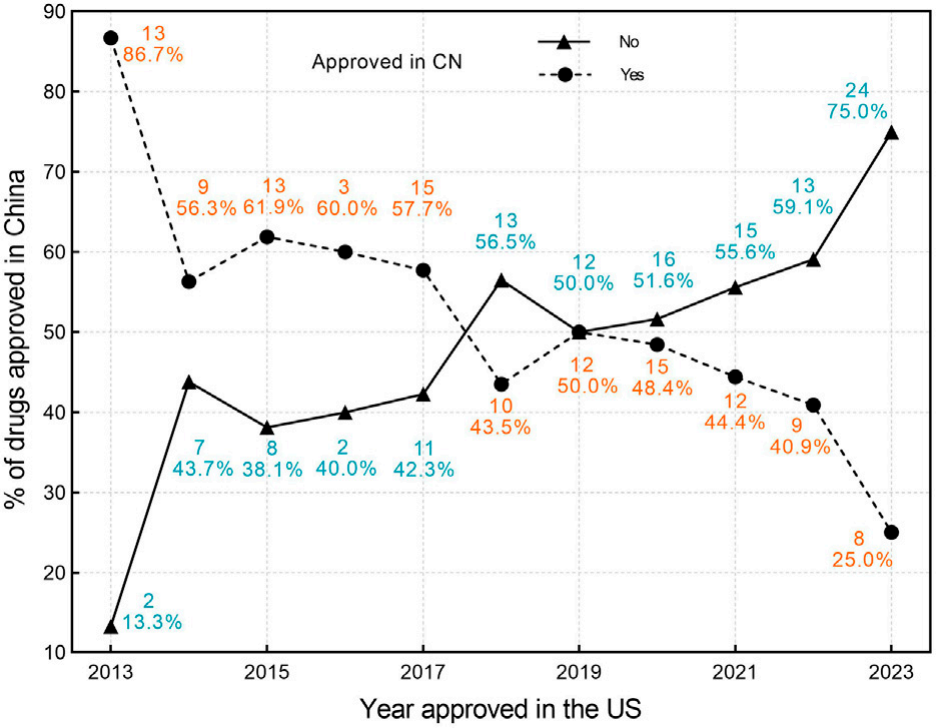
The drug lag in China for orphan drugs approved by the FDA in 2013–2023. Circles denote the number and proportion of drugs approved in China, while triangles denote the number and proportion of drugs not approved in China. CN, China; US, United States.

**FIGURE 3:**
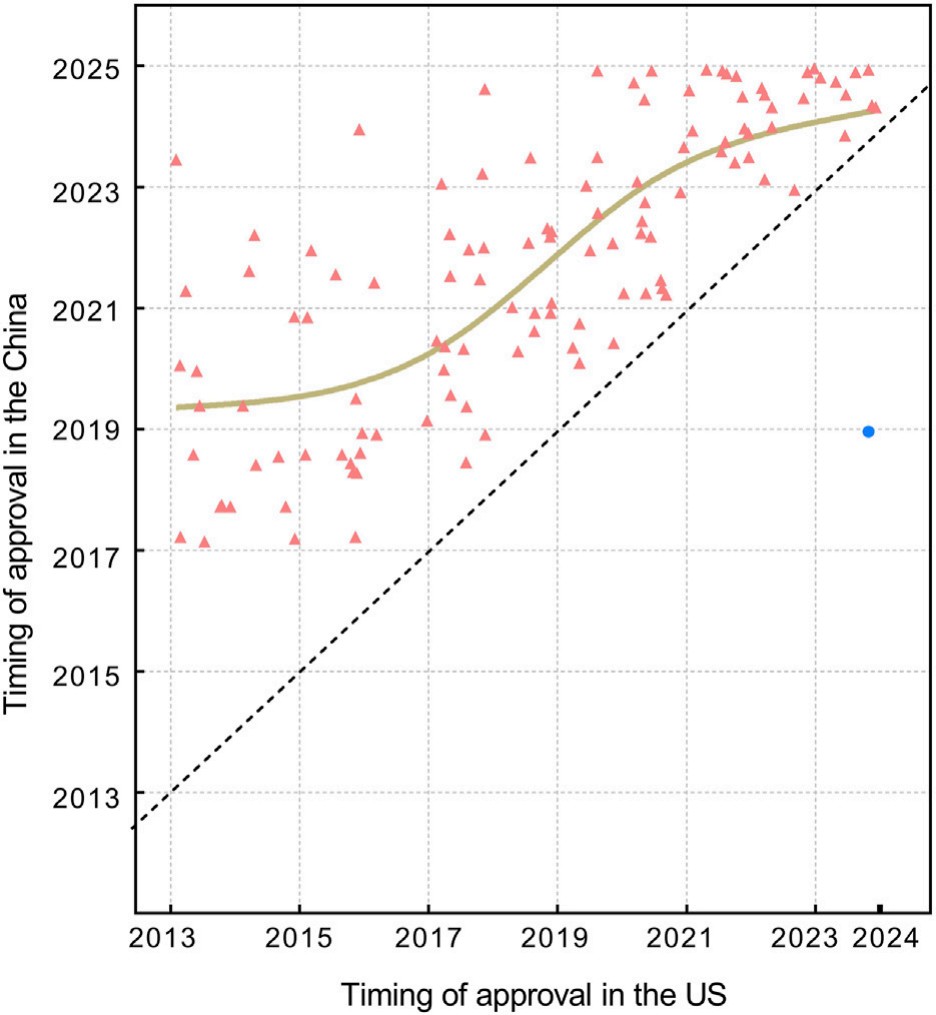
The market entry distribution in China for orphan drugs approved by the FDA in 2013–2023. Red triangles denote drugs first approved in the United States, while blue circles denote drugs first approved in China. The brown fitted line is constructed using the locally weighted scatterplot smoothing (LOWESS) method, indicating the trend of relative drug lag. The diagonal dashed line represents the case of no lag (i.e., simultaneous approval by the FDA and NMPA), and symbols or the fitted line that approach this diagonal line indicate shorter lag times.

Among the 119 drugs approved in China, the average lag time in marketing approval compared with the United States is 1,147 days (3.14 years), with a median lag time of 1,004 days (2.75years) (IQR,723-1,442), and a range of 103 to 3,785 days. Among these, only one drug was approved in China before the United States, with an advance time of 1,775 days (4.8 years). Figures 2, 3 illustrate the trend of drug lag changes over the years.

The absolute lag is severe among newly approved drugs at the FDA (Figure 2), while the relative lag shows an improving trend over time, as shown by the reduced gap between the fitted line and the oblique line in Figure 3. The gap between the fitted line and the diagonal line has narrowed, but the gap still exists.

### 3.2 Associations with the drug lag and related factors

The results of the multinomial logistic regression (Table 2) show that the registration category, therapeutic area, and drug indication have no significant impact on whether a drug is approved in China.

**TABLE 2:** Factors to the drug lag in China for orphan drugs approved by the FDA in 2013–2023.

Variable	OR	Robust SE	P value	95%CI
Type of drug				
Chemical drugs Biologics	1 [Reference] 0.80	0.458	0.490	0.44-1.48
Indication				
Non-cancer Cancer	1 [Reference] 1.21	0.351	0.572	0.61-2.41
Breakthrough therapy designation by the FDA				
No Yes	1 [Reference] 1.11	0.309	0.717	0.61-2.05
Accelerated approval by the FDA				
No Yes	1 [Reference] 1.39	0.366	0.364	0.68-2.85
China’s List of Rare Diseases				
No Yes	1 [Reference] 0.67	0.307	0.199	0.37-1.23
Pivotal Trial as MRCTs in FDA				
No Yes	1 [Reference] 2.16	0.430	0.063	0.96-5.17
MAH company size in the U.S.				
Large pharma Small to mid-sized enterprise	1 [Reference] 1.2	0.307	0.547	0.66-2.19
Year approved in the U. S.	0.844	0.05	**<0.001**	0.76-0.93
Pivotal trial locations in FDA				
No China sites	1 [Reference]			
With sites in China mainland	10.53	0.614	**<0.001**	3.67-40.79
With sites in Hongkong/Taiwan, China	1.50	0.41	0.302	0.69-3.33

However, the location of pivotal clinical trials supporting the FDA’s initial approval is correlated with whether these drugs are marketed in China. Drugs that conducted clinical trials in mainland China have a higher chance of obtaining approval in China (OR = 10.53, 95% confidence interval: 3.67-40.79; *P* < 0.001). This characteristic was not observed for drugs that conducted clinical trials in Hong Kong and/or the Taiwan region, China.

Meanwhile, there is a negative correlation between the year of FDA approval and whether the drug is marketed in China, indicating that drugs approved by the FDA more recently have a lower probability of being marketed in China. This also suggests that the absolute lag ratio of drugs in China is showing an increasing trend year by year. In addition, whether the FDA breakthrough therapy designation and accelerated approval were obtained did not show a significant correlation with drug approval in China.

### 3.3 Correlates of drug lag time and related factors

To study the reasons for the long lag time of orphan drugs in more detail, we used the nationality of the MAH and the types of companies., whether the pivotal clinical trial was an MRCT, pivotal clinical trial site, the therapeutic area of the disease, the CLRD, urgently needed overseas drugs (UNODs) by China, and the breakthrough therapy designation by the FDA and NMPA as independent variables (Table 3), then we calculated the median and interquartile range of the drug lag times, and used the Mann-Whitney U test to determine if there were significant differences between these variables.

**TABLE 3:** Factors to the drug lag in China for orphan drugs approved by the FDA in 2013–2023.

Independent variable	N (%)	Lag time (Median [IQR]) (Days)	P value^a^
Type of drug			
Chemical drugs	77 (64.7)	1,042 (711-1,493)	0.525
Biologics	42 (35.3)	962 (728-1,325)	
Oncology Drugs			
Yes	62 (52.1)	1,120 (802-1,491)	0.121
No	57 (47.9)	844 (635-1,416)	
Pivotal clinical trial in FDA			
MRCT	109 (91.6)	1,016 (706-1,416)	0.237
Non-MRCT	10 (8.4)	997 (834-2,110)	
China’s List of Rare Diseases			
Yes	47 (39.5)	811 (481-1,162)	**0.001**
No	72 (60.5)	1,211 (803-1,637)	
Urgently needed overseas drugs			
Yes	17 (14.3)	1,082 (791-1,922)	0.550
No	102 (85.7)	999 (714-1,435)	
Marketing authorization holder in the United States			
Large pharma	75 (63.0)	1,071 (766-1,495)	0.150
Small to mid-sized enterprise	44 (37.0)	869 (703-1,313)	
Marketing authorization holder in China			
Nationality			
China	8 (6.7)	805 (200-970)	**0.045**
Foreign-affiliated	111 (93.3)	1,042 (731-1,489)	
Company size			
Large pharma	85 (71.4)	1,071 (727-1,497)	0.286
Small to mid-sized enterprise	34 (28.6)	931 (713-1,293)	
Expedited pathway in FDA			
Breakthrough therapy			
Yes	64 (53.8)	984 (646-1,353)	0.154
No	55 (46.2)	1,035 (800-1,644)	
Accelerated approval			
Yes	41 (34.5)	1,035 (711-1,326)	0.675
No	78 (65.5)	999 (731-1,475)	
Expedited pathway in NMPA			
Breakthrough therapy			
Yes	23 (19.3)	602 (200-910)	**<0.001**
No	96 (80.7)	1,100 (803-1,597)	
Conditional Approval			
Yes	45 (37.8)	994 (706-1,310)	0.267
No	74 (62.2)	1,025 (748-1,509)	
Pivotal trial locations in FDA			
No China site	70 (58.8)	1,117 (779-1,601)	Ref.
With sites in China mainland^b^	31 (26.1)	917 (396-1,115)	**0.009**
With sites in Hongkong/Taiwan, China^b^	36 (30.3)	896 (467-1,129)	**0.010**

^a^ Mann-Whitney U test.

^b^ In multicenter clinical trials, research sites in Chinese mainland, Taiwan (China) and Hong Kong (China) may be included simultaneously.

We found that the lag time of drugs whose pivotal clinical trials included China mainland, Hong Kong/Taiwan, China as trial sites at the time of their first approval by the FDA was significantly shorter than that of drugs not including China as a clinical trial site. The lag times were 917 days [IQR,396–1,115], 896 days [IQR,467–1,129]and 1,117 days [IQR,779–1,601], respectively, which was also a significant difference (*P* < 0.05). This phenomenon may be related to the ‘Clinical Technical Requirements for Drugs Approved Overseas but Not Yet Approved in China’ issued by the NMPA in 2020. The document stipulates that if the data from overseas clinical trials cannot rule out racial sensitivity, additional bridging trials are required when the drug is launched in China.

Further analysis revealed that the time lag for drugs with indications included in the CLRD issued in China was significantly shorter than those listed in the CLRD (median 811 days [IQR,481-1,162] vs 1,211 days [IQR,803-1,637]) respectively, with a significant difference (*P* < 0.05).

We found that the approval dates for drugs whose MAH are Chinese companies are shorter than those for drugs from foreign companies. The median and interquartile range (IQR) for the two groups are 805 days [IQR, 200–970] and 1,042 days [IQR, 731–1,489] respectively, with a statistically significant difference (*P <* 0.05).

In addition, we observed a significant difference in the time lag between drugs that received NMPA breakthrough therapy designation and those that did not, indicating that it has a positive effect on the speed of market entry for drugs in China. However, factors such as the FDA’s breakthrough therapy designation, whether it is an oncology drug, the size of company, and the drug marketing license holder did not show a significant impact on the time lag to market in China in the statistical analysis.

Analysis of covariance identified significant factors associated with drug lag, including breakthrough therapy in the United States, breakthrough therapy in China, China’s rare disease list, and clinical trial sites in Mainland China (all P-values <0.05; Supplementary Table S1).

### 3.4 Development status of drugs not approved in China

Among the 242 orphan drugs approved by the FDA, a total of 123drugs have not yet been approved in China. Of these 15 (12.2%) are currently undergoing Marketing Authorization Applications (MAA) with the NMPA, 29 (23.6%) are in various stages of clinical trials, and the remaining 79 (64.2%) have no plans for submission or research in China. These undeveloped drugs may potentially lack development plans in China due to factors such as low prevalence in China, a small number of patients, insufficient expectations of market returns, as well as patients’ payment capacity and regulatory differences.

**FIGURE 4:**
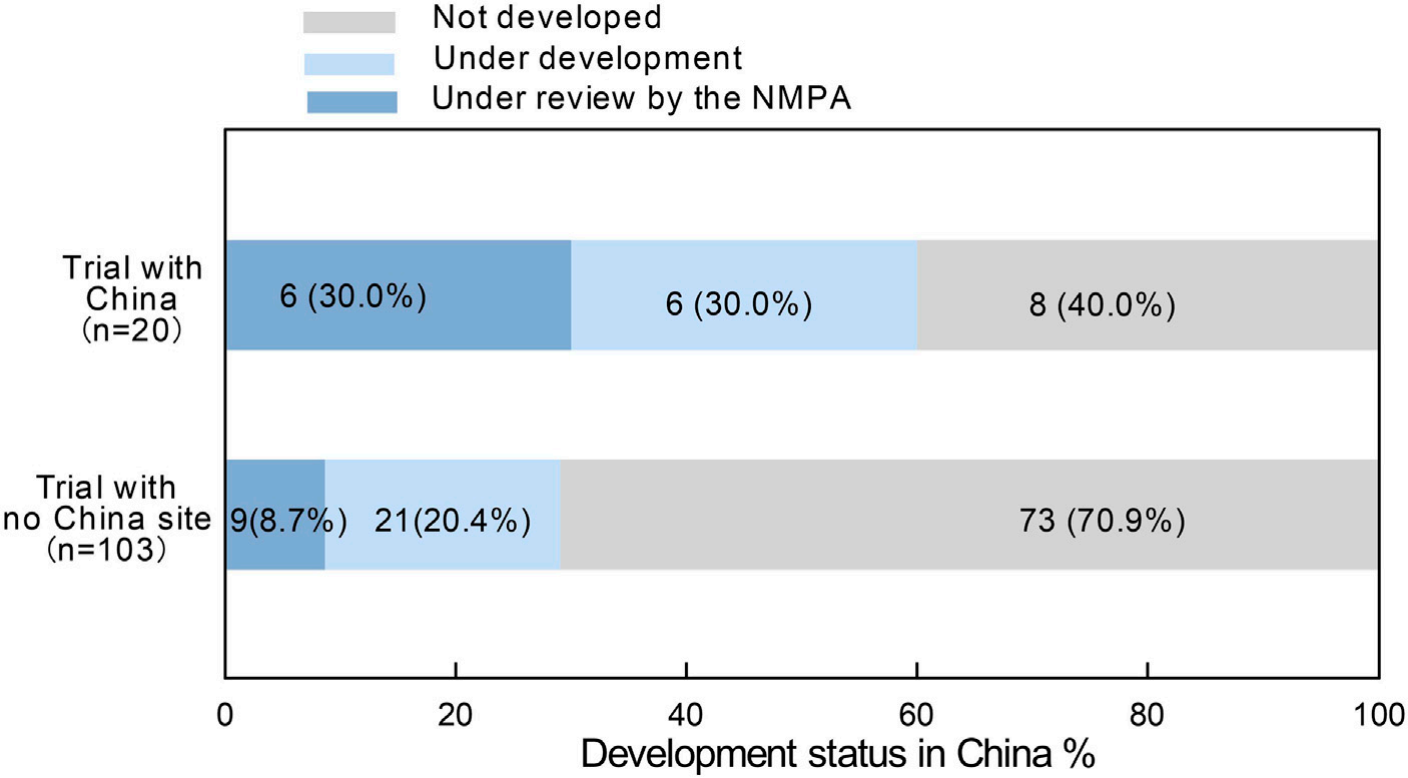
R&D status in China for orphan drugs approved in the United States but not approved in China. “Trail with china” includes Chinese mainland, Taiwan (China), and Hong Kong (China).

We reviewed the development status of these drugs in China as of 15 February 2025. Among the 20 drugs whose pivotal clinical trials included research centers in China (including Taiwan and Hong Kong), 30% are currently submitting MAA to the NMPA, 30% are in various stages of clinical trials, and 40% have not yet initiated further development in China. In contrast, among the 103 drugs that did not include China as a clinical trial site, only 8.7% are currently applying for marketing authorization with the NMPA, 20.4% are in various stages of clinical trials, and 70.9% have not yet undergone any development activities in China (Figure 4).

## 4 Discussion

Our work assessed the access gap of orphan drugs from the United States in China. Less than half of the orphan drugs approved by the FDA during 2013–2023 were licensed in China, with a median lag time of 1,004 days (2.75 years) and a maximum lag time of 3,785 days (10.4 years).

The measured result, compared with the recently published time lag of 3.5 years for new drugs approved by the FDA to enter the Chinese market from 2012 to 2019 (Zhu and Chen, 2024), shows a slight reduction in the time lag (2.75 vs 3.5 years), which may be attributed to the gradual improvement in the drug accessibility gap over time as well as the special status of orphan drugs.

According to relevant literature, the time lag between approving anti-cancer orphan drugs in Japan and the United States was 1.9 years from 2016 to 2017 (Nakayama, Matsumaru, and Tsukamoto, 2019). The time lag between the approval of new drugs in South Korea and the United States was 1.9–2.2 years from 2016 to 2019 (Cho and Han, 2022). Overall, the lag times in Japan and South Korea are similar. In contrast, there remains a significant gap in access to innovative orphan drugs between rare disease patients in China and those in developed countries. Delayed access to innovative drugs not only undermines patients’ health but also increases the opportunity cost of using inferior therapies. However, this lag has gradually improved over time.

Moreover, many Chinese patients with rare diseases are facing the predicament of “drugs available overseas but unavailable domestically,” which indicates that the country needs to introduce more policies to strive to narrow the gap in access to orphan drugs (Liu et al., 2023).

We found that whether a drug is marketed in China is mainly related to whether the Chinese mainland is included as a site in its pivotal clinical trials. These factors are associated with the target countries chosen by enterprises for marketing, expected market returns, and regulatory differences between countries. In recent years, the NMPA has been committed to facilitating the approval of new drugs already marketed overseas in China, strengthening international cooperation, and standardizing regulatory rules. This initiative aims to introduce more innovative and effective drugs into the Chinese market, benefiting Chinese patients (Chinese Government Website, 2018). For example, the Chinese government has released three batches of the “List of Clinically Urgently Needed Overseas Drugs,” calling on drugs included in the list to submit marketing applications in China as soon as possible. Drugs included in the list can undergo accelerated review through the priority review and approval pathway, and relevant studies have shown that this policy has played a significant role in accelerating drug approval (Wei et al., 2025).

In terms of relative lag time, drug lag time is mainly associated with whether the MAHs is a Chinese company, whether the Chinese mainland is included as a pivotal clinical trial site, whether the drug’s indication is listed in CLRD, and whether it has obtained breakthrough therapy designation from the NMPA.

In addition, other factors in clinical trial design significantly impact the drug approval process in China. For example, among the 119 drugs, 31 (26.1%) conducted pivotal trials supporting FDA approval in the Chinese mainland, and 36 (30.3%) in China’s Taiwan and Hong Kong regions. These drugs entered the Chinese market faster than those that did not conduct pivotal trials in China. This may be related to the inclusion of a larger number of Chinese participants in their early stages. For drugs without pivotal trials in China, the lack of data on Asian populations may require supplementary bridging clinical trials when applying for market approval in China, thereby delaying their entry into the Chinese market. In addition, this is also related to whether companies have sufficient confidence in the Chinese market and are willing to proactively enter it.

Therefore, we recommend that pharmaceutical companies consider including Chinese patients in the early stages of research and development. At the same time, we also hope that China will introduce more supportive policies to strengthen the confidence of foreign enterprises in entering the Chinese market.

The Breakthrough Therapy designation is intended to expedite the development and review of drugs for serious or life-threatening conditions. Whether a drug has been granted breakthrough therapy designation by the NMPA is an important factor affecting drug approval delays. Previous studies support our findings that breakthrough therapy designation drugs can reduce the time for clinical trials and reviews compared to non-breakthrough therapy designation drugs. Therefore, the breakthrough therapy designation drug development approach can significantly shorten the time for new drug development (Michaeli et al., 2024).

China’s breakthrough therapy designation applies to drugs that are used to prevent or treat diseases that seriously threaten life or severely affect quality of life, with no effective prevention or treatment methods available, or for which there is sufficient evidence showing significant clinical advantages compared with existing treatment methods. We believe that such drugs may be launched in China more quickly due to their urgent clinical needs and clinical advantages.

The CLRD has a total of 207 indications. A drug’s lag time is associated with whether its indication is included in this list. This may be attributed to China’s special policy support for orphan drugs, such as priority review and approval procedures and preferential status in national medical insurance access negotiation which have significantly boosted pharmaceutical companies’ enthusiasm for introducing such orphan drugs into China (Zhao et al., 2023; China NMPA, 2020).

Moreover, compared with off-CLRD indications, those included in the CLRD are associated with higher prevalence, a larger patient population, and thus a bigger market for the relevant drugs. These factors may affect the timing of applicants’ submission for marketing in China (Lu et al., 2022; Li et al., 2024).

In summary, therefore, to accelerate the launch of new drugs in China, it is recommended that pharmaceutical companies include Asian populations and even Chinese populations in their early R&D stages, or use China as one of the research sites for Multinational Clinical Trials (MRCTs). This measure may effectively shorten the drug approval cycle in China and, at the same time, provide important strategic support for enterprises’ global market layout.

We suggest that China should further strengthen its focus on orphan drugs, include more orphan drugs in the medical insurance system, and establish a more comprehensive support mechanism for rare diseases. Meanwhile, China should introduce targeted policies to enhance the attractiveness of the Chinese market to foreign enterprises, promote the accelerated entry of more effective drugs into China, and thereby effectively safeguard the treatment opportunities for patients with rare diseases.

This study has certain limitations. First, this study only focuses on orphan drugs approved by the U.S. FDA and thus may not fully cover all orphan drugs approved in China. However, considering that the FDA is the institution that adopts the largest number of orphan drugs globally, the drug samples it has approved are widely representative of the overall market. Therefore, the overall impact of this limitation on the study results should be relatively limited. Second, the prevalence of rare diseases in China was not included in this study, as the data on the prevalence of rare diseases in China is still incomplete, and it is currently difficult to obtain authoritative relevant data.

## 5 Conclusion

The accessibility gap between China and the United States is a challenge for China, particularly concerning orphan drugs, where the phenomenon of ‘drugs available overseas but not approved domestically’ persists. However, in recent years, the NMPA has been working to close this gap and has made significant progress. China’s participation in the simultaneous development of new drugs around the world and simultaneous filing of drug applications across borders will bring benefits to Chinese patients and help expand opportunities for global pharmaceutical innovators. However, achieving this goal will require additional efforts and collaboration among all relevant stakeholders.

## Data Availability

Publicly available datasets were analyzed in this study. This data can be found here: https://www.accessdata.fda.gov/scripts/opdlisting/oopd/index.cfm, https://www.fda.gov/drugs/novel-drug-approvals-fda/novel-drug-approvals-2021.
