# Supplementary material for: Drug lag and associated factors of orphan drugs approved by the U.S. in China
Source: Front Pharmacol. 2025 Aug 29;16:1595497. doi: 10.3389/fphar.2025.1595497 (PMC12425770; doi:10.3389/fphar.2025.1595497)
Supplement: Supplementary file 1 [file DataSheet1.pdf]

Table S1 Analysis of covariance to identify risk factors contributing to orphan drug lag time [a]

Independent variables	Coefficient	Standard error	95% confidence intervals		95% confidence intervals
			Lower	Upper	
**Registration class** – BLA (Vs NDA)	-34.1	119.4	-271.0	202.7	0.776
**Indication** – Cancer (Vs Non-cancer)	71.6	140.8	-207.5	350.9	0.612
**US MAH's company type -** large pharma (Vs Small to mid-sized enterprise)	-232.3	166.3	-562.1	97.4	0.165
**CN MAH's company type -**large pharma (Vs Small to mid-sized enterprise)	86.4	184.3	-279.1	452.0	0.64
**CN MAH Nationality-**China (Vs Foreign-affiliated)	306.9	260.8	-210.2	824.2	0.242
**US/Accelerated Approval** – Yes (Vs No)	125 .8	160.0	-191.4	443.1	0.433
**US/Breakthrough Therapy** – Yes (Vs No)	-248.6	123.5	-493.5	-3.6	**0.047**
**CN/Accelerated Approval** – Yes (Vs No)	-195.3	147.3	-487.5	96.9	0.188
**CN/Breakthrough Therapy** – Yes (Vs No)	-380.3	167.8	-713.0	-47.5	**0.025**
**China two batches rare disease catalogs** – Yes (Vs No)	-280.2	121.9	-522.0	-38.4	**0.024**
**Year approved in the U. S.-**2019 to 2023(Vs 2013 to 2018)	-594.4	121.4	- 835.2	-353.6	**<0.001**
**The FDA pivotal trial is an MRCT-Yes** (Vs No) [b]	-345.0	202.6	-746.9	56.8	0.092
**Trial type-** With sites in CN (Vs No China site)	-369.6	135 .4	-638.2	-101.0	**0.007**
**Trial type** -With sites in Hongkong/TW(CN), China (Vs No China site) [c]	102.8	160.7	-421.6	215.8	0.524

[a] Analyze which factors influence the lag time of pharmaceuticals in China, the scope of the study includes 119 drugs that have already been launched in China.

[b] MRCT: Multi-Regional Clinical Trial.

[c] Pivotal clinical trial location: CN: China mainland; TW(CN): Taiwan, China: Foreign: Refers to overseas or foreign countries. HK(CN): Hong Kong, China.

Table S2 Raw data of the study drug samples

[a] NDA: New Drug Application; BLA: Biologics License Application.

[b] Expedited Programs in FDA:PR: Priority Review; FT: Fast Track; AA: Accelerated Approval; BTD: Breakthrough Therapy Designation; Standard: Standard review.

[c] Expedited Programs in NMPA:PR: Priority Review; BTD Standard: Standard review; CA: Conditional Approval.

[d] China rare disease catalog: The National Health Commission of China released two batches of the Rare Disease Catalogue in 2018 and 2023

[e] MRCT: Multi-Regional Clinical Trial.

[f] Pivotal clinical trial location: CN: China mainland; TW(CN): Taiwan, China: Foreign: Refers to overseas or foreign countries. HK(CN): Hong Kong, China.

[g] Status of the drug in China: Approved (Approval Date);Under submission: Drug is under marketing application in China；CTA approved: Clinical trial approved in China；Undeveloped: Drug is undeveloped in China; Phase II: Drug is in Phase II clinical trials; Phase III: Phase III clinical trials.

Drug	Drug class [a]	Expedited Programs in FDA [b]	Expedited Programs in NMPA [c]	China rare disease catalog [d]	Pivotal clinical trial in FDA [f]			Approval date [g]	
					MRCT [e]	location	NCT ID	NMPA	FDA
Iptacopan	NDA	FT、CA、BTD	PR、BTD	Yes	Yes	CN TW(CN)	NCT04558918 NCT04820530	2024/04/24	2023/12/05
Repotrectinib	NDA	PR、FT、CA、BTD	PR、AA、BTD	No	Yes	CN TW(CN)	NCT03093116	2024/05/08	2023/11/15
Toripalimab	BLA	PR、FT、BTD	PR、AA、BTD	No	Yes	CN TW(CN)	NCT03581786	2018/12/17	2023/10/27
Vamorolone	NDA	FT	PR、BTD	Yes	Yes	Foreign	NCT03439670	2024/12/06	2023/10/26
DaxibotulinumtoxinA	BLA	Standard	Standard	No	Yes	Foreign	NCT03014635 NCT03014622	2024/11/22	2022/9/8

Efgartigimod PH20 SC	BLA	PR	PR、BTD	Yes	Yes	CN<br>TW(CN)	NCT04281472	2024/07/09	2023/06/20
Glofitamab	BLA	PR、FT、CA、BTD	PR、AA、BTD	No	Yes	TW(CN)	NCT03075696	2023/11/07	2023/06/15
Tofersen	NDA	PR、FT、CA	AA	Yes	Yes	Foreign	NCT03070119	2024/09/26	2023/04/25
Pirtobrutinib	NDA	PR、FT、CA	PR、AA	No	Yes	Foreign	NCT03740529	2024/10/22	2023/01/27
Mosunetuzumab	BLA	FT、CA、BTD	PR、AA	No	Yes	Foreign	NCT02500407	2024/12/17	2022/12/22
Mirvetuximab soravtansine	BLA	PR、FT、CA	PR、AA	No	Yes	Foreign	NCT04296890	2024/11/22	2022/11/14
Teclistamab	BLA	FT、CA、BTD	PR、AA、BTD	No	Yes	CN	NCT04557098<br>NCT03145181	2024/06/18	2022/10/25
Spesolimab	BLA	PR、BTD	PR、BTD	Yes	Yes	CN<br>TW(CN)	NCT03782792	2022/12/13	2022/09/01
trientine	NDA	Standard	PR	Yes	Yes	Foreign	NCT03539952	2023/12/29	2022/04/28
Mavacamten	NDA	BTD	PR、BTD	No	Yes	Foreign	NCT03723655	2024/04/24	2022/4/28
Ganaxolone	NDA	PR	PR	Yes	Yes	Foreign	NCT03572933	2024/07/09	2022/03/18
Upadacitinib	NDA	PR	PR、BTD	No	Yes	CN<br>TW(CN)	NCT02706873<br>NCT02706951<br>NCT02675426<br>NCT02629159<br>NCT02706847	2022/2/18	2019/8/16
Ciltacabtagene	BLA	BTD	PR、AA、	No	No	Foreign	NCT03548207	2024/08/20	2022/02/28

autoleucel			BTD						
Efgartigimod	BLA	FT	BTD	Yes	Yes	Foreign	NCT03669588	2023/06/30	2021/12/17
Budesonide	NDA	PR、CA	PR、AA、BTD	No	Yes	TW(CN)	NCT03643965	2023/11/21	2021/12/15
Maribavir	NDA	PR、BTD	PR、BTD	No	Yes	Foreign	NCT02931539	2023/12/19	2021/11/23
Ropeginterferon alfa/2b/njft	BLA	Standard	PR	Yes	No	Foreign	NCT01193699	2024/06/28	2021/11/12
Avacopan	NDA	Standard	Standard	Yes	Yes	Foreign	NCT02994927	2024/10/29	2021/10/07
Maralixibat	NDA	PR、BTD	PR	Yes	Yes	Foreign	NCT02160782	2023/05/29	2021/09/29
Belzutifan	NDA	PR	PR	Yes	Yes	Foreign	NCT03401788	2024/11/15	2021/08/13
Avalglucosidase alfa	BLA	PR、FT、BTD	PR	Yes	Yes	TW(CN)	NCT02782741 NCT01898364 NCT02032524 NCT03019406	2023/09/28	2021/08/06
Odevixibat	NDA	PR、FT	PR	Yes	Yes	Foreign	NCT03566238	2024/12/01	2021/07/20
belumosudil	NDA	PR、BTD	BTD	No	No	Foreign	NCT03640481	2023/08/01	2021/07/16
Loncastuximab tesirine	BLA	PR、FT、CA	PR、AA	No	Yes	Foreign	NCT03589469	2024/12/06	2021/04/23
Tepotinib	NDA	PR、CA	Standard	No	Yes	CN TW(CN)	NCT02864992	2023/12/05	2021/02/03
Trastuzumab deruxtecan	BLA	PR、FT、CA、BTD	PR、AA、BTD	No	Yes	Foreign	NCT03248492 NCT02564900	2023/2/21	2019/12/15
Margetuximab	BLA	FT	Standard	No	Yes	Foreign	NCT02492711	2023/08/29	2020/12/16
Naxitamab	BLA	FT、CA、	PR、AA	Yes	Yes	Foreign	NCT03363373	2022/11/30	2020/11/25

		BTD					NCT01757626		
Pralsetinib	NDA	FT、CA、BTD	PR、AA、BTD	No	Yes	CN TW(CN)	NCT03037385	2021/03/23	2020/09/04
Satralizumab	BLA	FT、BTD	PR	Yes	Yes	TW(CN)	NCT02073279 NCT02028884	2021/04/30	2020/08/14
Risdiplam	NDA	PR、FT	PR	Yes	Yes	CN	NCT02913482 NCT02908685	2021/06/16	2020/08/07
Lurbinectedin	NDA	PR、CA	PR、AA	No	Yes	Foreign	NCT02454972	2024/12/01	2020/06/15
Inebilizumab	BLA	BTD	PR	Yes	Yes	TW(CN) HK(CN)	NCT02200770	2022/03/08	2020/06/11
Ripretinib	NDA	PR、FT、BTD	PR、AA	Yes	Yes	Foreign	NCT03353753	2021/03/30	2020/05/15
Selpercatinib	NDA	FT、CA、BTD	PR、AA	No	Yes	TW(CN) HK(CN)	NCT03157128	2022/09/30	2020/05/08
Capmatinib	NDA	FT、CA、BTD	Standard	No	Yes	TW(CN)	NCT02414139	2024/06/11	2020/05/06
Sacituzumab govitecan	BLA	PR、FT、CA、BTD	PR、AA	No	No	Foreign	NCT01631552	2022/06/07	2020/04/22
Pemigatinib	NDA	FT、CA、BTD	PR、AA	No	Yes	TW(CN)	NCT02924376	2022/03/29	2020/04/17
Durvalumab	BLA	FT、CA、BTD	Standard	No	Yes	CN TW(CN)	NCT03043872	2019/12/6	2017/5/1
Ozanimod	NDA	PR	Standard	Yes	Yes	Foreign	NCT02294058 NCT02047734	2023/01/31	2020/03/25
Osilodrostat	NDA	Standard	PR	No	Yes	CN	NCT02180217	2024/09/19	2020/03/06

Avapritinib	NDA	PR、FT、BTD	PR、AA	Yes	Yes	Foreign	NCT02508532	2021/03/30	2020/01/09
Zanubrutinib	NDA	PR、FT、CA、BTD	PR、AA、BTD	Yes	Yes	CN	NCT03206970<br>NCT02343120	2020/6/2	2019/11/14
Luspatercept	BLA	PR、FT	PR、AA	Yes	Yes	CN<br>TW(CN)	NCT02706873<br>NCT02706951<br>NCT02675426<br>NCT02629159<br>NCT02706847	2022/01/25	2019/11/08
Entrectinib	NDA	FT、CA、BTD	PR、AA	No	Yes	CN<br>TW(CN)	NCT02097810<br>NCT02568267<br>NCT02650401	2022/07/26	2019/08/15
pitolisant	NDA	PR、FT、BTD	PR	Yes	Yes	Foreign	NCT 01067222<br>NCT01638403<br>NCT01800045	2023/06/30	2019/08/14
Pretomanid	NDA	PR、FT	Standard	No	No	Foreign	NCT02333799	2024/12/01	2019/08/14
Selinexor	NDA	PR、FT、CA	PR、AA	No	Yes	Foreign	NCT02336815	2021/12/14	2019/07/03
Polatuzumab vedotin	BLA	FT、CA、BTD	PR、AA	No	Yes	Foreign	NCT02257567	2023/01/10	2019/06/10
Tafamidis meglumine	NDA	PR、FT、BTD	PR	Yes	Yes	Foreign	NCT01994889	2020/02/05	2019/05/03
Tafamidis	NDA	Standard	PR	Yes	Yes	Foreign	NCT01994889	2020/09/30	2019/05/03
Siponimod	NDA	PR	PR	Yes	Yes	CN	NCT01665144	2020/05/07	2019/03/26
gilteritinib	NDA	PR、FT	PR、AA	No	Yes	TW(CN)	NCT02421939	2021/01/30	2018/11/28

Larotrectinib	NDA	FT、CA、BTD	PR、AA	No	Yes	CN	NCT02122913<br>NCT02637687<br>NCT02576431	2022/04/08	2018/11/26
Venetoclax	NDA	FT、CA、BTD	PR、AA	No	Yes	Foreign	NCT01889186<br>NCT02203773	2020/12/02	2016/4/11
Emapalumab	BLA	PR、FT、BTD	PR、AA	No	Yes	Foreign	NCT01818492	2022/03/08	2018/11/20
Lorlatinib	NDA	FT、CA、BTD	PR、AA、BTD	No	Yes	TW(CN)<br>HK(CN)	NCT01970865	2022/04/27	2018/11/02
Lanadelumab	BLA	PR、FT、BTD	Standard	Yes	Yes	Foreign	NCT02586805	2020/12/02	2018/08/23
Cenegermin	BLA	PR、FT、BTD	Standard	Yes	Yes	Foreign	NCT05133180<br>NCT02227147	2020/08/12	2018/08/22
lusutrombopag	NDA	PR、FT	Standard	No	Yes	Foreign	NCT02389621	2023/06/27	2018/07/31
Ivosidenib	NDA	PR、FT、BTD	PR、AA	No	Yes	Foreign	NCT02074839	2022/01/30	2018/07/20
Avatrombopag	NDA	PR	PR	No	Yes	CN<br>TW(CN)	NCT01972529<br>NCT01976104	2020/04/14	2018/05/21
Burosumab	BLA	PR、FT、BTD	PR、AA	Yes	Yes	Foreign	NCT02163577	2021/01/05	2018/04/17
Emicizumab	BLA	PR、BTD	PR	Yes	Yes	TW(CN)	NCT02622321<br>NCT02795767	2018/11/30	2017/11/16
Benralizumab	BLA	Standard	Standard	No	Yes	Foreign	NCT01928771<br>NCT01914757<br>NCT02075255	2024/08/13	2017/11/14

letermovir	NDA	PR、FT、BTD	Standard	No	Yes	Foreign	NCT02137772	2021/12/31	2017/11/08
Acalabrutinib	NDA	FT、CA、BTD	AA	No	Yes	Foreign	NCT02213926	2023/03/21	2017/10/31
Axicabtagene ciloleucel	BLA	BTD	PR、AA、BTD	No	Yes	Foreign	NCT02348216	2021/06/22	2017/10/18
Inotuzumab ozogamicin	BLA	PR、BTD	PR	No	Yes	CN TW(CN)	NCT01564784	2021/12/20	2017/08/17
Glecaprevir+Pibrentasvir	NDA	PR、FT、BTD	PR、AA	No	Yes	CN	NCT02939989	2019/05/15	2017/08/03
Nivolumab	BLA	PR、FT、CA、BTD	PR、AA、BTD	No	Yes	Foreign	NCT04099251	2018/06/15	2014/12/22
Neratinib	NDA	Standard	Standard	No	Yes	CN TW(CN)	NCT00878709	2020/04/28	2017/07/17
Edaravone	NDA	Standard	PR	Yes	No	Foreign	NCT00424463 NCT01492686	2019/07/25	2017/05/05
Brigatinib	NDA	FT、CA、BTD	Standard	No	Yes	Foreign	NCT02094573	2022/03/22	2017/04/28
Deutetrabenazine	NDA	PR、BTD	PR	Yes	Yes	Foreign	NCT01795859	2020/05/12	2017/04/03
Niraparib	NDA	PR、FT、BTD	PR、AA	No	Yes	Foreign	NCT01847274	2019/12/26	2017/03/27
Ribociclib	NDA	PR、BTD	Standard	No	Yes	TW(CN)	NCT01958021	2023/01/19	2017/03/13
brodalumab	BLA	Standard	AA	No	Yes	Foreign	NCT02786732	2020/06/17	2017/02/15
Nusinersen	NDA	PR、FT	PR	Yes	Yes	Foreign	NCT02193074	2019/02/22	2016/12/23

Melphalan	NDA	Standard	PR	No	No	Foreign	NCT01660633	2018/11/30	2016/03/10
Obinutuzumab	BLA	PR、BTD	PR	No	Yes	Foreign	NCT01059630	2021/06/01	2013/11/1
Selexipag	NDA	Standard	PR	Yes	Yes	CN TW(CN)	NCT01106014	2018/12/07	2015/12/21
Alectinib	NDA	FT、CA、BTD	PR、BTD	No	Yes	CN TW(CN)	NCT03456076	2018/08/12	2015/12/11
Methylphenidate sustained/release chewable tablet	NDA	Standard	PR	No	No	Foreign	NCT01654250	2023/12/13	2015/12/04
Ixazomib	NDA	PR	PR	No	Yes	CN	NCT01564537	2018/04/12	2015/11/20
Daratumumab	BLA	PR、FT、CA、BTD	PR、AA	No	Yes	Foreign	NCT01985126	2019/07/04	2015/11/16
Osimertinib	NDA	PR、FT、CA、BTD	PR、BTD	No	Yes	TW(CN)	NCT01802632 NCT02094261	2017/03/22	2015/11/13
Glycopyrrolate	NDA	Standard	Standard	No	Yes	Foreign	NCT00929110	2018/04/16	2015/10/29
Idarucizumab	BLA	FT、CA、BTD	PR	No	Yes	TW(CN)	NCT02104947	2018/06/08	2015/10/16
evolocumab	BLA	PR	PR	Yes	Yes	HK(CN)	NCT01516879 NCT01588496 NCT01624142	2018/7/31	2015/08/27
Sonidegib	NDA	Standard	PR	No	Yes	Foreign	NCT01327053	2021/07/20	2015/07/24
isavuconazonium	NDA	PR	Standard	No	Yes	CN	NCT00412893	2021/12/14	2015/03/06
Lenvatinib	NDA	FT、CA、BTD	PR、AA	No	Yes	Foreign	NCT01321554	2018/9/4	2015/02/13

Palbociclib	NDA	FT、CA、BTD	PR	No	Yes	Foreign	NCT00721409	2018/07/31	2015/02/03
Ruxolitinib	NDA	PR	PR	Yes	Yes	CN	NCT01243944	2017/03/10	2014/12/4
Blinatumomab	BLA	FT、CA、BTD	PR、AA	No	Yes	Foreign	NCT01466179	2020/11/09	2014/12/03
Nintedanib	NDA	PR、FT、BTD	PR	Yes	Yes	CN	NCT01335464	2017/09/20	2014/10/15
Pembrolizumab	BLA	FT、CA、BTD	PR、AA	Yes	Yes	Foreign	NCT01295827	2018/07/20	2014/09/04
Ceritinib	NDA	FT、CA、BTD	PR	No	Yes	Foreign	NCT01283516	2018/05/31	2014/04/29
Ramucirumab	BLA	PR、FT	Standard	No	Yes	TW(CN)	NCT00917384	2022/03/16	2014/04/21
Apremilast	NDA	Standard	PR	No	Yes	Foreign	NCT01232283<br>NCT01690299	2021/08/12	2014/03/21
Elosulfase Alfa	BLA	PR、FT	PR	Yes	Yes	TW(CN)	NCT01275066	2019/05/21	2014/02/14
Sofosbuvir	NDA	PR、FT、BTD	PR	No	Yes	Foreign	NCT01682720	2017/09/20	2013/12/06
Macitentan	NDA	Standard	PR	Yes	Yes	CN	NCT01474109<br>NCT01474122<br>NCT01743001	2017/09/29	2013/10/18
Riociguat	NDA	PR	PR	Yes	Yes	CN<br>TW(CN)	NCT00910429<br>NCT00855465	2017/09/20	2013/10/08
Afatinib	NDA	PR、FT	PR	No	Yes	CN<br>TW(CN)	NCT00949650<br>NCT01121393<br>NCT00525148	2017/02/21	2013/07/12

Denosumab	BLA	PR	PR、AA	Yes	No	Foreign	NCT00896454	2019/05/21	2013/6/13
Trametinib	NDA	PR、FT、CA、BTD	PR、AA	Yes	Yes	Foreign	NCT01584648	2019/12/18	2013/05/29
Dabrafenib	NDA	PR、FT、CA、BTD	PR、AA	Yes	Yes	Foreign	NCT01584648	2019/12/18	2013/05/29
Fluticasone+Vilanterol	NDA	PR	Standard	No	Yes	Foreign	NCT01134042	2018/07/31	2013/05/10
Dimethyl fumarate	NDA	Standard	PR	Yes	Yes	Foreign	NCT00420212	2021/04/13	2013/03/27
Regorafenib	NDA	PR、FT	PR	Yes	Yes	CN	NCT01271712	2017/03/22	2013/2/25
Trastuzumab Emtansine	BLA	PR、FT、BTD	PR	No	Yes	TW(CN)、HK	NCT00829166	2020/01/21	2013/02/22
Glycerol phenylbutyrate	NDA	FT	PR	No	No	CN、TW(CN)	NCT00992459	2023/06/14	2013/02/01
Pomalidomide	NDA	PR、FT、CA、BTD	NA	No	Yes	Foreign	NCT01495598	Phase Ⅲ	2013/02/08
Prothrombin Complex Concentrate	BLA	Standard	NA	No	Yes	Foreign	NCT00803101	Undeveloped	2013/04/29
tasimelteon	NDA	PR	NA	No	No	Foreign	NCT00708435	Undeveloped	2014/1/31
Droxidopa	NDA	PR、FT、CA	NA	Yes	No	Foreign	NCT01428661	Undeveloped	2014/02/18
Metreleptin	BLA	PR、FT	NA	No	No	Foreign	NCT00633880 NCT00782340	Undeveloped	2014/02/24
miltefosine	NDA	PR、FT	NA	No	Yes	Foreign	NCT00819234	Undeveloped	2014/03/19
Belinostat	NDA	PR、FT、CA	NA	Yes	Yes	Foreign	NCT00378495	Undeveloped	2014/07/03

Eliglustat	NDA	PR	NA	Yes	Yes	CN	NCT00865969	Phase Ⅲ	2014/08/19
Cobicistat	NDA	PR、FT	NA	No	Yes	Foreign	NCT00943111 NCT01074944	Undeveloped	2014/9/24
Ivacaftor+Lumacaftor	NDA	PR、FT、BTD	NA	Yes	Yes	Foreign	NCT01108510	Undeveloped	2015/7/2
Uridine triacetate	NDA	PR、FT、BTD	NA	No	No	Foreign	NCT01807923 NCT01807949	Undeveloped	2015/9/4
Asfotase alfa	BLA	PR、FT、BTD	NA	Yes	Yes	Foreign	NCT02110147	Undeveloped	2015/10/23
trabectedin	NDA	PR	NA	No	Yes	Foreign	NCT01176266	Phase Ⅲ	2015/10/23
talimogene laherparepvec	BLA	Standard	NA	Yes	Yes	Foreign	NCT01343277	Undeveloped	2015/10/27
Cobimetinib	NDA	PR、FT、BTD	NA	Yes	Yes	Foreign	NCT00769704	Undeveloped	2015/11/10
Elotuzumab	BLA	PR、BTD	NA	No	Yes	Foreign	NCT01689519	Undeveloped	2015/11/30
Sebelipase alfa	BLA	PR、FT、BTD	NA	Yes	Yes	Foreign	NCT01757184	Undeveloped	2015/12/08
Eteplirsen	NDA	PR、FT、CA	NA	Yes	No	Foreign	NCT01757184	Undeveloped	2016/09/19
Rucaparib	NDA	FT、CA、BTD	NA	No	Yes	Foreign	NCT02255552	Undeveloped	2016/12/19
Deflazacort	NDA	PR、FT	NA	Yes	No	Foreign	NCT02952534 NCT01482715	Undeveloped	2017/02/09
Telotristat etiprate	NDA	PR、FT	NA	No	Yes	Foreign	NCT01335295 NCT01603407	Undeveloped	2017/02/28

Avelumab	BLA	PR、FT、CA、BTD	NA	Yes	Yes	Foreign	NCT02063659	Phase Ⅲ	2017/03/23
Valbenazine	NDA	PR、FT、BTD	NA	Yes	Yes	Foreign	NCT02684006	Undeveloped	2017/04/11
Cerliponase Alfa	BLA	PR、BTD	NA	No	Yes	Foreign	NCT02274558<br>NCT04102579	Undeveloped	2017/04/27
Midostaurin	NDA	PR、FT、BTD	NA	Yes	Yes	Foreign	NCT02678689	CTA approved	2017/04/28
Enasidenib	NDA	PR、FT	NA	No	No	Foreign	NCT00651261	Phase Ⅲ	2017/08/01
Tisagenlecleucel	BLA	CA、BTD	NA	No	Yes	Foreign	NCT01915498	Phase Ⅲ	2017/08/30
Vestronidase alfa	BLA	PR、FT	NA	Yes	No	Foreign	NCT02445248<br>NCT02435849	Undeveloped	2017/11/15
Voretigene neparvovec	BLA	BTD	NA	Yes	No	Foreign	NCT02230566	Undeveloped	2017/12/19
Macimorelin	NDA	Standard	NA	Yes	Yes	Foreign	NCT00999609	Undeveloped	2017/12/20
Tezacaftor+Ivacaftor	NDA	PR、FT、BTD	NA	Yes	Yes	Foreign	NCT02558829	Undeveloped	2018/02/12
Fostamatinib	NDA	FT	NA	No	Yes	Foreign	NCT02347657<br>NCT02392234<br>NCT02516410	Undeveloped	2018/04/17
Pegvaliase	BLA	PR、FT	NA	Yes	No	Foreign	NCT02076399<br>NCT02076399<br>NCT02076412	Undeveloped	2018/05/24
Cannabidiol	NDA	PR、FT	NA	Yes	Yes	Foreign	NCT01819727<br>NCT01889862	Undeveloped	2018/06/25
Binimetinib	NDA	Standard	NA	Yes	Yes	Foreign	NCT02224560	Phase II	2018/06/27

							NCT02224690<br>NCT02091375<br>NCT02091375		
Tecovirimat	NDA	PR、FT	NA	No	No	Foreign	NCT01909453	Undeveloped	2018/07/13
Tafenoquine	NDA	PR、BTD	NA	No	Yes	Foreign	NCT04971109	Undeveloped	2018/07/20
PATISIRAN	NDA	PR、FT、BTD	NA	Yes	Yes	TW(CN)	NCT01376167	Undeveloped	2018/08/10
migalastat	NDA	PR、FT、CA	NA	Yes	Yes	Foreign	NCT01960348	Undeveloped	2018/08/10
stiripentol	NDA	PR	NA	Yes	No	Foreign	NCT00925301	Undeveloped	2018/08/20
Glasdegib	NDA	PR	NA	No	Yes	Foreign	NCT03819647	Phase Ⅲ	2018/11/21
Ravulizumab	BLA	PR	NA	Yes	Yes	TW(CN)	NCT01546038	Under submission	2018/12/21
Tagraxofusp	BLA	PR、BTD	NA	No	No	Foreign	NCT02946463<br>NCT03056040	Undeveloped	2018/12/21
Caplacizumab	BLA	PR、FT	NA	No	Yes	Foreign	NCT02113982	Undeveloped	2019/02/06
Triclabendazole	NDA	PR、FT	NA	No	Yes	Foreign	NCT02553317<br>NCT01151423	Undeveloped	2019/02/13
Solriamfetol	NDA	Standard	NA	Yes	Yes	Foreign	NCT04230148	Under submission	2019/03/20
Onasemnogene abeparvovec	BLA	BTD	NA	Yes	No	Foreign	NCT01722539	Phase Ⅲ	2019/05/24
Alpelisib	NDA	FT、CA、BTD	NA	Yes	Yes	TW(CN)、HK（CN）	NCT02348593<br>NCT02348606<br>NCT02348619	Phase Ⅲ	2019/05/24

							NCT02348632<br>NCT01681121		
Pexidartinib	NDA	PR、BTD	NA	Yes	Yes	Foreign	NCT03306277<br>NCT03505099	Under submission	2019/08/02
Fedratinib	NDA	PR	NA	Yes	Yes	TW(CN)	NCT02437318	Phase Ⅲ	2019/08/16
Afamelanotide	BLA	PR、FT	NA	Yes	Yes	Foreign	NCT02371369	Undeveloped	2019/10/08
Ivacaftor+Tezacaftor+ Elexacaftor	NDA	PR、FT、BTD	NA	Yes	Yes	Foreign	NCT01437787	Undeveloped	2019/10/21
Diroximel fumarate	NDA	Standard	NA	Yes	Yes	Foreign	NCT 01605136<br>NCT00979745<br>NCT01097044	Phase Ⅲ	2019/10/29
Givosiran	NDA	PR、BTD	NA	Yes	Yes	TW(CN)	NCT03525444<br>NCT03525548	Undeveloped	2019/11/20
Golodirsen	NDA	PR、FT、CA	NA	Yes	Yes	Foreign	NCT02634307	Undeveloped	2019/12/12
Teprotumumab	BLA	PR、FT、BTD	NA	No	Yes	Foreign	NCT03338816	CTA approved	2020/01/21
Tucatinib	NDA	PR、FT、CA、BTD	NA	No	Yes	Foreign	NCT02310906<br>NCT0250381	Phase Ⅲ	2020/04/17
Triheptanoin	NDA	FT	NA	No	No	Foreign	NCT01868997<br>NCT03298867	Undeveloped	2020/06/30
Cedazuridine+Decitabine	NDA	PR	NA	No	No	Foreign	NCT02614794	Phase II	2020/07/07
Brexucabtagene autoleucel	BLA	BTD	NA	No	Yes	Foreign	NCT018863<br>NCT022141<br>NCT01379625	Phase II	2020/07/24

Tafasitamab	BLA	PR、FT、CA、BTD	NA	No	Yes	Foreign	NCT02103478 NCT03306264	Under submission	2020/07/31
Nifurtimox	NDA	PR、CA	NA	No	Yes	Foreign	NCT02614066 NCT02601313	Undeveloped	2020/08/06
Viltolarsen	NDA	PR、FT、CA	NA	Yes	No	Foreign	NCT02399085	Under submission	2020/08/12
lonafarnib	NDA	PR、BTD	NA	Yes	Yes	Foreign	NCT02625974	Undeveloped	2020/11/20
Lumasiran	NDA	PR、BTD	NA	No	Yes	Foreign	NCT02740972	Undeveloped	2020/11/23
Setmelanotide	NDA	PR、BTD	NA	Yes	Yes	Foreign	NCT00425607 NCT00916747	CTA approved	2020/11/25
Berotralstat	NDA	FT	NA	Yes	No	Foreign	NCT03681184 NCT03905694	Undeveloped	2020/12/03
Lisocabtagene maraleucel	BLA	FT、CA、BTD	NA	No	No	Foreign	NCT02896192 NCT03287960	Undeveloped	2021/02/05
Evinacumab	BLA	PR、BTD	NA	Yes	Yes	Foreign	NCT03485911	Undeveloped	2021/02/11
Casimersen	NDA	PR、FT、CA	NA	Yes	Yes	Foreign	NCT03331198 NCT03575351	Undeveloped	2021/02/25
Fosdenopterin	NDA	PR、BTD	NA	No	Yes	Foreign	NCT03399786	Undeveloped	2021/02/26
Pegcetacoplan	NDA	PR、FT	NA	Yes	Yes	Foreign	NCT03532542	Undeveloped	2021/05/14
Sotorasib	NDA	PR、FT、CA、BTD	NA	No	Yes	TW(CN)	NCT02047461	Phase Ⅲ	2021/05/28
Brincidofovir	NDA	PR、FT	NA	No	Yes	Foreign	NCT03500549	Undeveloped	2021/06/04
Plasminogen	BLA	Standard	NA	No	No	Foreign	NCT05198934 NCT03600883	Undeveloped	2021/06/04

Asparaginase Erwinia chrysanthemi	BLA	FT	NA	No	Yes	Foreign	NCT01769170	Undeveloped	2021/06/30
fexinidazole	NDA	PR	NA	No	Yes	Foreign	NCT02690714	Undeveloped	2021/07/16
Lonapegsomatropin	BLA	Standard	NA	Yes	Yes	Foreign	NCT04145531	Under submission	2021/08/25
Rethymic	BLA	BTD	NA	No	No	Foreign	NCT01685827	Undeveloped	2021/10/08
Asciminib	NDA	PR、FT、CA、BTD	NA	No	Yes	Foreign	NCT02781727	Under submission	2021/10/29
Vosoritide	NDA	PR、CA	NA	Yes	Yes	Foreign	NCT00566488 NCT00576836	Undeveloped	2021/11/19
Pafolacianine	NDA	PR、FT	NA	No	No	Foreign	NCT02081378	Undeveloped	2021/11/29
Tebentafusp/tebn	BLA	PR、BTD	NA	Yes	Yes	Foreign	NCT03583697	Undeveloped	2022/01/25
Sutimlimab	BLA	PR、BTD	NA	Yes	Yes	Foreign	NCT03180307	Undeveloped	2022/02/04
Mitapivat	NDA	PR、FT	NA	No	Yes	Foreign	NCT03070392	Undeveloped	2022/02/17
Pacritinib	NDA	PR、FT、CA	NA	Yes	Yes	Foreign	NCT03347422	Undeveloped	2022/02/28
Nivolumab+Relatlimab	BLA	PR、FT	NA	Yes	Yes	Foreign	NCT03548220 NCT03559699	Phase Ⅲ	2022/03/18
Vutrisiran	NDA	PR、FT	NA	Yes	Yes	TW(CN)	NCT02055781	Undeveloped	2022/06/13
Olipudase alfa	BLA	PR、FT、BTD	NA	Yes	Yes	Foreign	NCT03470922	Undeveloped	2022/08/31
Futibatinib	NDA	PR、FT、CA、BTD	NA	No	Yes	HK（CN）	NCT03759379	CTA approved	2022/09/30
Tremelimumab	BLA	PR	NA	No	Yes	CN	NCT02004691	Under	2022/10/21

						HK（CN）	NCT02292654 NCT02004704	submission	
Etranacogene dezaparvovec	BLA	PR、BTD	NA	Yes	Yes	Foreign	NCT02052778	Undeveloped	2022/11/22
Rebyota	BLA	FT、BTD	NA	No	No	Foreign	NCT03298451 NCT03164616	Undeveloped	2022/11/30
Olutasidenib	NDA	Standard	NA	No	Yes	Foreign	NCT03569891	Undeveloped	2022/12/01
Adagrasib	NDA	PR、FT、CA、BTD	NA	No	Yes	Foreign	NCT03244644	Phase Ⅲ	2022/12/12
Sparsentan	NDA	PR、CA	NA	No	Yes	TW(CN)、HK（CN）	NCT02719574	Undeveloped	2023/02/17
Omaveloxolone	NDA	PR、FT	NA	Yes	Yes	Foreign	NCT03785249	Undeveloped	2023/02/28
Trofinetide	NDA	PR、FT	NA	Yes	No	Foreign	NCT03762850	Undeveloped	2023/03/10
Retifanlimab	BLA	PR、FT、CA	NA	Yes	No	Foreign	NCT02255435	Phase Ⅲ	2023/03/22
Rezafungin	NDA	PR、FT	NA	No	Yes	TW(CN)	NCT04181723	Phase Ⅲ	2023/03/22
Leniolisib	NDA	PR	NA	Yes	Yes	Foreign	NCT04472429 NCT03599713	Undeveloped	2023/03/24
Omidubicel	BLA	PR、BTD	NA	No	Yes	Foreign	NCT03667690	Undeveloped	2023/04/17
Epcoritamab	BLA	FT、CA、BTD	NA	No	Yes	Foreign	NCT02435173	Under submission	2023/05/19
Delandistrogene moxeparvovec	BLA	PR、CA	NA	Yes	No	HK（CN）	NCT02730299	Undeveloped	2023/06/22

Rozanolixizumab	BLA	PR	NA	Yes	Yes	TW(CN)	NCT03625037	Under submission	2023/06/26
Valoctocogene Roxaparvovec	BLA	PR、BTD	NA	Yes	Yes	TW(CN)	NCT05096221	Undeveloped	2023/06/29
quizartinib	NDA	PR、FT、BTD	NA	No	Yes	CN HK （CN）	NCT03971422	Under submission	2023/07/20
Elranatamab	BLA	PR、FT、CA、BTD	NA	No	Yes	Foreign	NCT03370913	Under submission	2023/08/14
Palovarotene	NDA	PR、BTD	NA	Yes	Yes	Foreign	NCT02668653	CTA approved	2023/08/16
Pozelimab	BLA	PR、FT	NA	Yes	No	Foreign	NCT04649359	Phase Ⅲ	2023/08/18
Motixafortide	NDA	Standard	NA	No	Yes	Foreign	NCT03312634	Phase Ⅲ	2023/09/08
Momelotinib	NDA	Standard	NA	Yes	Yes	TW(CN)	NCT04209634	Undeveloped	2023/09/15
Cipaglucosidase alfa	BLA	BTD	NA	Yes	Yes	TW(CN)	NCT03246529	Undeveloped	2023/09/28
Nedosiran	NDA	BTD	NA	No	Yes	Foreign	NCT01969838 NCT04173494	Undeveloped	2023/09/29
Zilucoplan	NDA	Standard	NA	Yes	Yes	Foreign	NCT03729362 NCT02675465 NCT04138277	Under submission	2023/10/17
Nirogacestat	NDA	PR、FT、BTD	NA	No	Yes	Foreign	NCT03847909 NCT04042402	Undeveloped	2023/11/27
Lovotibeglogene autotemcel	BLA	PR、FT	NA	Yes	No	Foreign	NCT04115293	Undeveloped	2023/12/08
Exagamglogene	BLA	PR、FT	NA	Yes	Yes	Foreign	NCT03785964	Undeveloped	2023/12/08

autotemcel									
Eplontersen	NDA	FT	NA	Yes	Yes	TW(CN)	NCT02140554<br>NCT04293185	Under submission	2023/12/21
Ansuvimab	BLA	PR、BTD	NA	No	No	Foreign	NCT03745287<br>NCT03655678	Undeveloped	2020/12/21
64Cu/DOTATATE	NDA	PR、FT	NA	No	No	Foreign	NCT04136184<br>NCT01737398	Undeveloped	2020/09/03
